# Perceived Race Affects Configural Processing but Not Holistic Processing in the Composite-Face Task

**DOI:** 10.3389/fpsyg.2018.01456

**Published:** 2018-08-20

**Authors:** Michael B. Lewis, Peter J. Hills

**Affiliations:** ^1^School of Psychology, Cardiff University, Cardiff, United Kingdom; ^2^Department of Psychology, Bournemouth University, Poole, United Kingdom

**Keywords:** face processing, own-race bias, holistic processing, configural processing, composite-face effect

## Abstract

One explanation for the own-race bias in face recognition is the loss of holistic processing for other-race faces. The composite-face task (involving matching the top halves of faces when the bottom halves are either changed or the same) tests holistic processing but it has been inconsistent in revealing other-race effects. Two composite-face experiments are reported using pairs of faces that have common internal features but can be perceived as either being racially Black or White depending on their external features. In Experiment 1 (matching the top halves of faces) holistic processing was found for both face races for White participants (shown by both a mis-alignment advantage when bottom halves were different and also by a congruence-by-alignment interaction in discrimination). Bayesian analysis supported there being no effect of race. However, the size of the simple congruence effect was larger for own- than for other-race faces. Experiment 2 found that this race-by-congruence interaction was not present when matching the bottom halves of faces. The results are interpreted in of terms of the perceived race affecting the processing of second-order relational information rather than holistic processing.

## Introduction

Faces of people from a race that a person is less familiar with tend to be recognized more poorly than faces from a race that they are familiar with. This is the own-race bias (or cross-race effect): Meissner and Brigham (2001) provide a meta-analysis of this phenomenon illustrating that it occurs across a variety of categories that are interpreted conceptually as races. There are many explanations for this own-race bias (see Hugenberg et al., 2010 for a review). Possible explanations include that social pressures mean that out-groups are not encoded as well as in-groups (Rodin, 1987) or that there are differences in the way other-race faces vary physiognomically, which make them more difficult to encode (Valentine and Endo, 1992). The question that is asked here is how are faces of an unfamiliar race processed differently from those of a familiar race.

Faces are typically processed configurally (e.g., Sergent, 1984) or holistically (e.g., Tanaka and Farah, 1993) although the exact difference between these terms is difficult to pin down and is discussed further below. It has been suggested that the difference between own-race face processing and other-race face processing is that the former employs more holistic processing than the later (e.g., Tanaka et al., 2004) and equally, configural encoding has been shown to play a role in the own-race bias by Rhodes et al. (2006) – although, they also show that race affects part-based processing of faces too and so the exact interaction between race and processing styles is complicated.

The focus of the current study is to evaluate whether there is a difference in the degree of holistic processing for own-race and other-race faces. We begin by discussing holistic processing and its relationship with configural processing. It is discussed how the composite-face illusion has been used to evaluate holistic processing and then the effects of race on the size of the composite-face effect are considered. In order to address the issue of whether race affects style of processing, it is important to be able to change the perceived race of faces without changing the specific visual properties being assessed in the task. We present two experiments using a novel stimuli set in which the perceived race of the faces could be manipulated while keeping the changes to the configurations of the face constant. The research aimed to evaluate whether the perceived race of a face affects the degree of holistic processing employed while keeping the features being assessed constant between the races.

### Holistic Processing and the Composite-Face Illusion

The concept of configural processing has been influential in understanding face recognition. Holistic processing is one type of configural processing as defined by Maurer et al. (2002) – the others being first-order and second-order relations between features. There are a number of definitions of holistic processing in face processing (see for example, Farah et al., 1998; McKone et al., 2003; Rossion, 2008). Richler et al. (2012) provide a detailed insight into the various meanings of holistic processing indicating that the mechanism could be based on global templates or spatial relations between features. Arguably, if holistic processing is just the spatial relations between features then it is the same as second-order relations as defined by Maurer et al. (2002) and so, here, the term holistic processing is used to refer to just global templates whereas second-order relational processing is considered separately. First-order configural information is the standard relative position of features within a face and it has been shown that holistic processing can still occur where the first-order configuration is biologically impossible (de Heering et al., 2012). It is unlikely that these configural processing features are related to the simple ratios of vertical and horizontal distances as Sandford and Burton (2014) have shown that people are poor at reproducing the correct aspect ratios of faces.

Holistic processing of faces is believed to be responsible for a range of effects including the whole-face advantage (Tanaka and Farah, 1993), the face-inversion effect (Yin, 1969), the composite-face illusion (Hole, 1994), and the Thatcher illusion (Thompson, 1980). Further, holistic processing is believed to be central to expertise in face recognition. As the face-inversion effect is reportedly smaller for other-race faces than for own-race faces (Sangrigoli and de Schonen, 2004) then this could be indicative that there is a difference in holistic encoding between the two types of faces with less holistic encoding being used for other-race faces.

It has been argued that the composite-face effect is a direct measure of holistic processing (see Rossion, 2013, or Murphy et al., 2017, for reviews). This effect is based on Young et al. (1987) composite-face illusion. This illusion is the demonstration that changing the bottom half of a face made the top half difficult to recognize unless the halves are misaligned. Hole’s (1994) extended the illusion into a task that involved matching the top halves of unfamiliar faces. Since then, there have been many studies that have made use of the composite-face effect to investigate the presence or absence of holistic processing.

In a typical experiment, such as those by Michel et al. (2007), two faces are presented one after another and either the top half of the face is the same or they are different and the participant is required to respond as to whether they are the same or different. Importantly, the bottom half of the face is different between the two images. Also, the two images may be constructed normally or there may be a misalignment between the top and the bottom of the two faces. A composite-face effect, as described by Rossion (2013), is the faster and/or more accurate performance for saying that two identical top halves of faces are the same in the misaligned condition compared to when the faces are aligned into a whole face. This difference between the misaligned and aligned conditions is used as evidence of holistic processing because in the aligned condition the presence of the different bottom half of the face creates a whole that is different from original and those whole impacts on one’s ability to evaluate just the top half of the face.

The size of the composite-face effect has been shown to be moderated by a range of variables and hence it has been suggested that holistic processing is disrupted by these manipulations. For example, Gao et al. (2011) showed that looking at the local elements of Navon (1977) letters disrupted holistic encoding and so might explain the Navon effect on face recognition tasks (Macrae and Lewis, 2002; Lewis et al., 2009). Further, rotation in the picture plane beyond 60° decreased the composite-face effect (Mondloch and Maurer, 2008; Rossion and Boremanse, 2008) which is consistent with holistic processing being greatly reduced for inverted faces.

While the face-composite effect can reveal holistic processing, it has been demonstrated that there is more to configural face processing than just the holistic processing. This was demonstrated because the individual differences in the size of the composite-face effect did not correlate with the sizes of the face-inversion effects or the effects in a whole-part face processing task (Rezlescu et al., 2017). This provides evidence that holistic processing may not be the whole story when it comes to understanding configural processing of faces. This idea is similar to a conclusion drawn by Hayward et al. (2013), who in their review concluded that different face tasks are assessing different types of configural information and holistic information is just one of these types.

### Race and the Composite-Face Illusion

Importantly for the current study, the composite-face task has been used to investigate holistic processing across races. Michel et al. (2006) used the task with Asian and White faces and with participants who were either Asian or White. A composite-face effect was found for White faces with White and Asian participants but for Asian faces it was only found for Asian participants. The significant three-way interaction revealed that the composite-face effect was larger for own-race faces than for other-race faces. The conclusion, therefore, was that holistic processing is reduced for other-race faces.

It could be argued that Michel et al. (2006) results provide a robust answer to whether there are different processing styles for own- and other-race faces. However, there is a confound between the features being evaluated and the perception of race. As well as switching the style of processing from own-race to other-race, the study also changed the features in the top half of the face from own-race to other-race. Evidence for this being a real issue can be seen most clearly in the White participants’ data. What is observed in the data is that the performance on the Asian aligned faces is equivalent to the performance on the White aligned faces – that is, with holistic processing for both races. There is a significant difference between performance on the misaligned Asian faces and the misaligned White faces (neither of which should have holistic encoding). A loss of holistic encoding, therefore, cannot be the whole story for the own-race bias as performance is worse for other-races in a matching task that does not employ holistic processing. An alternative explanation for Michel et al. (2006) results is that performance is worse for matching of other-race features. The disappearance of the composite-face effect for Asian faces with White participants could be a floor effect such that the loss of holistic processing does not have any further detrimental effect. In this case, holistic processing could still be employed for other-race faces but this would not be apparent in the data. It is worth noting that this explanation can also be applied to similar findings looking at the composite-face effect in own-age faces and other-age faces (de Heering and Rossion, 2008).

In order to correct for the feature-and-race confound it would be necessary to test comparisons of the same features but with different racial contexts. That is the same images are tested but they are either being processed as same-race or as other-race faces by the same people. This is what Michel et al. (2007) did using racially ambiguous faces. Images that were the perceptual midpoint in a White-to-Asian morph continuum were selected and used as critical items in a composite-face task (the midpoint was assessed by White participants as these made up the participants of the main experiment). These racially ambiguous items were either presented among a series of White faces or a series of Asian faces hence providing a racial context for the images. The research found a face-composite effect for both White and Asian faces. For the racially ambiguous faces, however, the face-composite effect was larger in the White (own-race) context than in the Asian (other-race) context suggesting a race effect on holistic processing.

While being a clever design, Michel et al. (2007) experiment does not completely resolve the issue. The critical effect, the difference in composite-face effect between the racial contexts, was only marginally significant “*t*(48)_one-tailed_ = 1.77” (p917). Indeed, Bayesian analysis of the critical effect (see more details below) suggests that the data provide anecdotal evidence for the null hypothesis that the composite effect is the same in both contexts rather than evidence for a difference (Bayes factor*_p_*_(hypothesis/nullhypothesis)_ = 0.658). As such, this provides less than weak evidence that the composite-face effect is reduced in faces interpreted as being of another race.

A follow-up study used the same stimuli but used an adaption procedure to help provide the racial context to the racially ambiguous faces (Michel et al., 2010). A 16 s exposure of an Asian face was used to make the racially ambiguous face appear White and vice versa. Over two experiments, a racial effect on the composite-face effect was found in accuracy but not reaction times in Experiment 1 but in reaction times but not accuracy in Experiment 2 which had corrected a possible demand characteristic present in the former experiment. Given that these reported effects were only just significant, it is unlikely that they would have survived a Bonferroni correction (Dunn, 1961) from having tested both reaction times and accuracies. As such, the findings remain inconclusive.

A summary of the studies on the composite-face effect in other-race faces is that where the features are matched between the racial contexts, there is only weak statistical evidence for a difference in the size of the composite-face effect for faces perceived as other-race faces. As well as being statistically inconclusive, some would argue that the design of these experiments was incomplete.

### Congruence Effects

The discussion on the composite-face effect has, so far, only considered the original version of the paradigm. Gauthier and Bukach (2007) argued that this represents only part of what is needed to understand holistic face processing. In the original or standard design, which they refer to as a partial design, the same top of a face is always paired with a different bottom and so there is an overall incongruence of response (‘same’ from top – ‘different’ from bottom). In the ‘different’ condition the bottom is also different and so there is a congruence of response (‘different’ from top – ‘different’ from bottom). In this way, the response decision is confounded with the congruence of the two parts of two stimuli. Also, there is the possibility that response bias may be influencing the findings.

Gauthier and Bukach (2007) argue that the composite-face effect should be assessed using a complete design in which both levels of correct response are required for both congruent and incongruent stimuli. The additional conditions required are, therefore, pairs where the tops are the same and the bottoms are the same (same-and-congruent) and pairs where the tops are different but the bottoms are the same (different-and-incongruent). According to Bukach et al. (2006), face expertise can be assessed by comparing performance in the congruent trials with performance in the incongruent trials. Performance is assessed using a signal detection calculation for the detection of the top halves being the same with false positives coming from incorrect responses to different-top-halves trials. Holistic face processing is concluded if performance on congruent trials is better than performance on incongruent trials. This holistic processing congruence effect disappears when the faces are misaligned (Richler et al., 2011a) showing that congruence is limited to intact or whole faces.

Rossion (2013) argues that this congruence analysis is not the correct analysis to address the composite-face effect because it is only assessing response bias. These arguments have been extensively discussed by Richler and Gauthier (2013, 2014). However, there are variations in how the complete method is employed. Many studies compare the size of the congruence effect for aligned and misaligned faces (for example, Richler et al., 2011b) whereas others just measure the size of the congruence effect for aligned faces (for example, Richler et al., 2009b; Wyer et al., 2015). Richler and Gauthier (2014) suggest that comparison with the misaligned conditions are important for determining holistic processing as congruence effects that are not modulated by misalignment have been observed for novices in music notation experiments (Richler et al., 2009a).

This complete method of analysis has been used to address whether other-race faces are processed holistically. First, Bukach et al. (2012) looked at the degree of social contact people had with other-race faces when assessing the effect that race had on the composite-face effect. Black and White participants were tested on Black and White face composites. Using reaction times rather than accuracy they showed that these measures were unaffected by the race of the faces being seen – hence no race effect on holistic processing. Further, race did not interact with the effects of congruence or alignment of faces (although the *p*-value for Black participants was 0.056). However, the size of the congruence advantage (faster responses when the tops matched and the bottoms matched or the tops were different and the bottoms were different) was reported to be larger in other-race faces when the participant had had more contact with other-race faces. As such, if only those who had low contact with other races were tested then it would be expected that a race effect on the size of congruence effect would be seen. As such, there is no clear evidence for an effect of race on holistic encoding but also this is not clear evidence that there is no effect of race on holistic encoding.

A second study, by Harrison et al. (2014), looked at the own-race bias effects in the composite face illusion using the congruence-by-alignment interaction as a measure of holistic processing. This study found holistic processing for both own-race faces and other-race faces for both White and Asian faces. There was a non-significant (*p* = 0.22) in-group advantage in holistic processing as indicated by a larger congruency-by-alignment interaction for in-group faces. This study clearly demonstrates the presence of holistic processing in other-race faces but it is possible that there is a sub-significant effect of race on the size of the holistic processing.

Lastly, the holistic processing of other-race faces was supported by Horry et al. (2015). They found holistic face processing for both Asian and European faces when processed by both Asian and European participants as indicated by the interactions between congruence and alignment. These interactions between congruence and alignment were unaffected by the race of the participants or the faces viewed with race effect sizes being very close to zero. This provides evidence that the processing of other-race faces is just as holistic as the processing of own-race faces.

In summary, analysis of the effect of race on holistic processing using the complete method of analysis suggests that other-race faces are processed holistically and there is some suggestion that the size of this holistic processing is equivalent to that for own-race faces. However, it is difficult to demonstrate equivalence of two effects although Bayesian methods of analysis are beginning to be used to test for the absence of an effect and will be used below. These methods were employed here to address the issue of whether there is a difference or equivalence in the size of the holistic processing for other-race and own-race faces.

## The Present Study

The aim of the current research was to use a task similar to the composite-face task to assess the difference in the degree of holistic processing for own-race and other-race faces regardless of the features being processed. In doing this, it was desirable to maintain the internal features as being as similar as possible between races while only changing their perceived race. The reason for doing this was that the aim was to isolate the effect of the perception of the race of the face separate from the changes to the internal features that are related to the race of a face. The question being asked is whether seeing a face as being other-race is sufficient to shift the nature of processing from holistic to non-holistic even when the features being assessed are identical.

MacLin and Malpass (2001, 2003) demonstrated that when the features of a face are suitably racially ambiguous, the perceived race of a face can be strongly manipulated by the hairstyle alone. The images generated by MacLin and Malpass (2001, 2003) had an obviously artificial feel to them, but they did demonstrate that it is possible to make faces whose internal features are constant but external features provide different yet convincing racial identities. Updated versions of these race-interchangeable faces were generated for the experiments described here. Using these stimuli, we assessed the extent of holistic processing in a composite-face task for face seen as either own- or other-race while keeping the size of the visual change the same between races.

The analysis of the data employed both the standard method (an analysis of alignment effects for incongruent same trials) advocated by Rossion (2013) and the complete method advocated by Richler and Gauthier (2013, 2014). While the complete model is normally assessed by looking at congruence-by-alignment interactions, configural encoding can also be assessed by looking at just congruence effects. Both of these versions of the complete analysis were assessed here.

### Generation of Race-Interchangeable Faces

**FIGURE 1:**
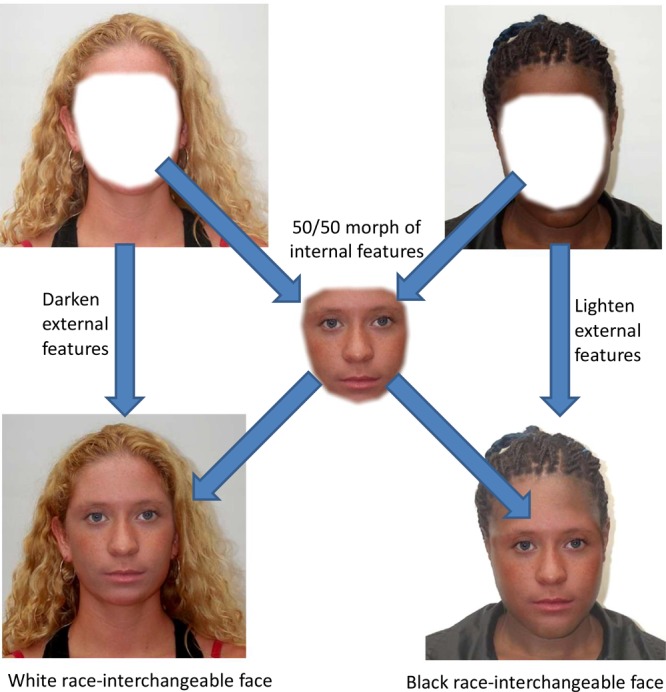
Construction of the race-interchangeable faces. A White and a Black parent faces are morphed to produce a blended image of the internal features. These internal features are inserted back into the parent faces whose external features have been either lightened or darkened so that the skin lightness matches the morph.

Six White and six Black female faces (aged between 18 and 30 years old) with neutral expressions were selected from the Minear and Park (2004) database. As much as possible, these faces were selected such that they were representative of the White and Black racial category [that is, faces that may have been mixed race were avoided – see Lewis (2016), for a discussion on this issue]. Pairs of one Black face and one White face were selected and blended together using morphing software to generate a 50% image (Tiddeman et al., 2001). The internal features (eyes, nose, mouth, and surrounding areas) of the 50% morphs were cropped. The parent face images were either lightened or darkened so that the internal features were of a similar color to the 50% morph. The morphed internal features were then pasted into the parent faces (see **Figure 1**).

**FIGURE 2:**
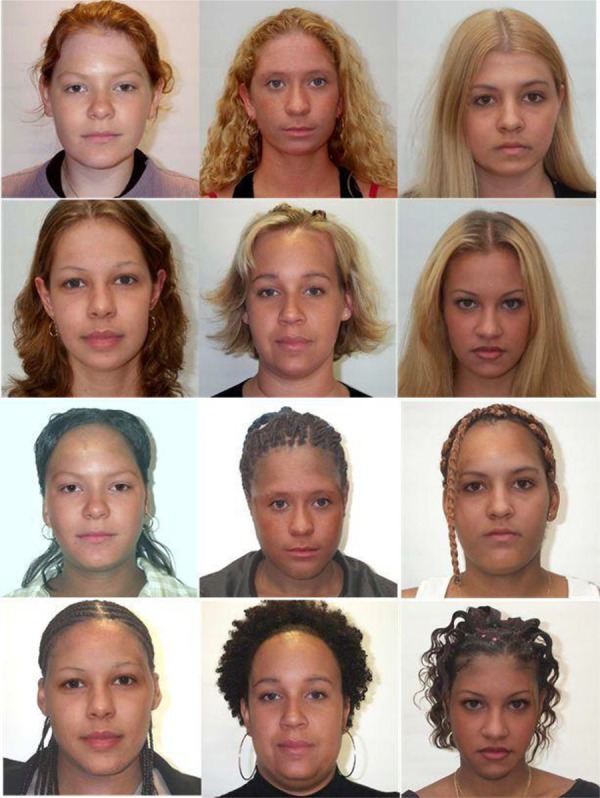
The six pairs of race-interchangeable faces. Pairs have identical internal features but different external features leading to a perception of different races between the pairs.

The resulting images were six faces that appeared to be ethnically White and six faces that appeared to be ethnically Black (see **Figure 2**) while their internal features were exactly matched between the races. Pilot testing (six White participants and three Black participants) confirmed this racial categorisation of the faces to be 87% accurate. The pilot participants also did not see the resemblances between the pairs of images. This is similar to the finding that people did not notice that Clinton’s internal features were placed on Gore’s face in Sinha and Poggio’s (1996) illusion. In fact, it was often necessary to occlude the external features for the participant to be convinced that the internal features were the same in the pairs. While these pairs of faces are perceived as being of different races, they also have identical internal features. The eyes, nose and mouth of each pair is the same. As such, identical changes can be made to faces from each racial group and any difference in processing of the images will be down to the racial interpretation of the images rather than the features themselves. This makes a useful set of images for testing whether the processing of facial features is difference because of the perception of them being of another race or because faces of other races vary in different ways. For example, in an analysis by Carroll and Chang (1970), the lower half of the internal features accounted for 75% of the variance in Black faces but only 35% of the variance in White faces. The current stimuli normalize these changes between the different classes of faces.

One advantage of using these stimuli is that it reduced the necessity to run a fully crossed design with participants from both racial categories. The reason for using a crossed design is that there may be some feature of the other-race or own-race faces that lead to a particular result that would not occur if the own-race faces were other-race and the other-race faces were own race (e.g., maybe the eyes are less variable in one race that the other). Using the race-interchangeable faces mean that the key features of the stimuli are controlled across races so same sets of eyes are effectively seen as both own-race and other-race. This is the same argument as offered by Michel et al. (2007) when using race-ambiguous faces in their composite-face task.

## Experiment 1

Experiment 1 assessed holistic processing using a task that is similar to the composite-face task but using the race-interchangeable faces. In this task, participants judged whether the top half of a face was altered while the bottom half of the face was either altered or not. A full design was employed with all combinations of similarity between top and bottom halves of the faces. Misalignment of the face parts was also included as a factorial condition.

There were a few differences between the current experiment and the standard composite-face task. First, rather than introducing a completely new top half and a new bottom half, the difference to be spotted was a small difference in the position of the eyes and the difference to be ignored was a small difference in the position of the mouth. This has the advantage of maximizing the chance that holistic processing is being employed in the task as the isolated features remain constant in everything except position. The task cannot be done using feature-based analysis of the images.

A second difference is that whole head stimuli are used. One advantage of this is that the images are more natural looking and hence are more likely to be processed in a natural and expert manner. Further, this resolves the issue of whether to ovalise faces or not. Richler et al. (2011a), for example, crop their faces to a common oval. Rossion (2013) criticizes this practice as it leads to poor alignment of faces and a reduction in the size of the composite-face effect. The usual alternative is to crop roughly to the hairline (e.g., Michel et al., 2010) but this leads to variation in the shape of the cropping. These variations in shape would be an extra-facial clue that could be used by participants to make a same/different decision. The solution of the whole head being used here means that there is no artificial cropping of the image and there is always a good match between the top and bottom half of the image.

This revised version of the composite-face task was used to test the relative use of holistic processing in faces that were perceived as own- or other-race. It was hypothesized that there will be a composite-face effect such that a change in the position of the mouth will lead to a decrease in performance in the determining that the eyes are the same or different. Further, the hypothesis being tested was that this composite-face effect will be moderated by the perceived race of the face such that it is smaller for other-race faces – even though the sizes of the changes to the images are equated. Bayesian analysis was used to test evidence for the hypotheses relative to their null hypotheses in order to establish evidence for equivalence between conditions.

### Method

#### Participants

There were 64 participants. Seven participants were not included in the data as they did not report the White faces as being of the same race as themselves (these participants were East Asian or South Asian). A further three participants were removed as they reported that the majority of the people where they grew up were of a different ethnicity to themselves. This left 54 participants who were aged 18 to 22 years, were White and who grew up in majority White communities.

#### Stimuli

**FIGURE 3:**
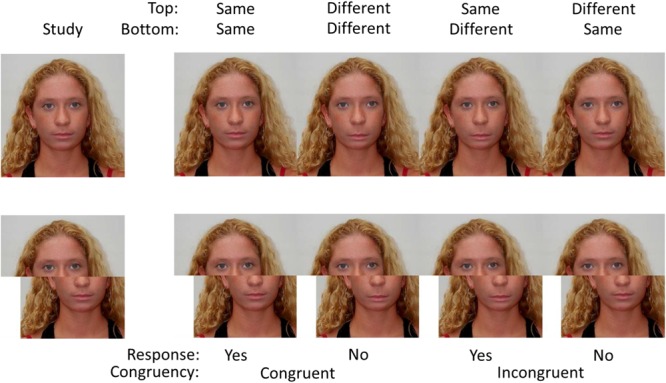
Examples of trials in Experiment 1. The task was to determine if the eyes are the same when the eyes and/or the mouth may have been changed. The top images show the aligned faces whereas the bottom images show the misaligned images.

Each of the original 12 race-interchangeable faces, described above, had the position of their eyes and/or mouth moved to generate 36 extra images. Twelve new images were created by increasing or decreasing the distance between the eyes of the faces. The images were created such that the one image had eyes that were 15% further apart than the other image (according to norms collected by Farkas et al., 2005, a change of 16.4% from the average in the distance between the insides of the eyes represents 1 standard deviation from that average). A further 12 images were generated by moving the position of the mouth up or down. The vertical height of top lip (base of the nose to the mouth opening) in the face with the lower mouth was approximately 50% larger than height of the top lip in the face with the higher mouth. The remaining 12 faces were constructed such that both changes were present. A further 48 images were generated by splitting the images along the middle of the nose and moving the lower half of the face to the right such that the nose was to the right of the right eye. Faces that were race-interchangeable pairs were always altered in exactly the same way. **Figure 3** shows a selection of the images generated in this way.

#### Procedure

**FIGURE 4:**
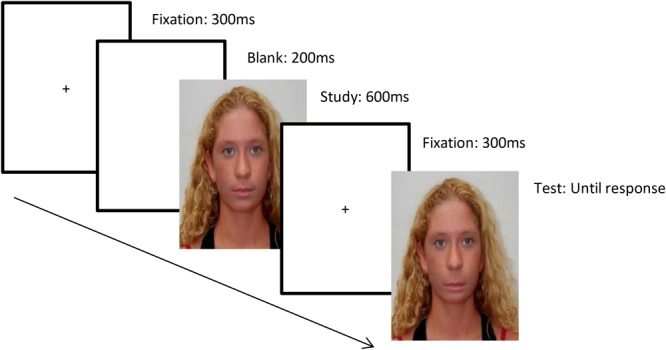
An example of a trial. In this example the top halves of the two faces are the same but the bottom halves are different. The correct response would be ‘same’ for Experiment 1.

After demographic details were collected, participants were presented with a series trials consisting of pairs of faces. The structure of each trial was as follows: there was a 300 ms fixation cross which then disappeared for 200 ms; a study face appeared for 600 ms followed by a fixation cross for 300 ms, and finally there was a test face that was presented until a response was made (see **Figure 4**). The participants’ task was to indicate whether the eyes were identical in the study face and the test face using two keys on a keypad. They were encouraged to respond as quickly and as accurately as possible. The accuracy of the responses was recorded using DirectRT.

There were 384 trials during the experiment. In each trial, the study face and the test face were of the same individual but each of the four versions of the face (two eye positions by two mouth positions) was paired with all four versions. This meant that there were 16 comparison pairs, half of which had the same eyes meaning that the correct response would have been ‘same’ (although half of these pairs would have had different mouths). There were a further 16 comparison pairs for each face based on the first set but constructed such that the face at test and study was the split (or misaligned) version. These 32 comparison pairs were generated for each of the 12 race-interchangeable faces making the 384 trials in the experiment.

Following the eye-change-decision task, the 12 original race-interchangeable images were presented one at a time. For each image, the participant was required to indicate whether they thought that the person would consider themselves as having the same ethnicity as the participant or a different ethnicity. A filter debrief was employed, which involved asking participants whether they thought the faces looked unusual in any way and whether they saw any similarities between the faces. This was carried out to assess whether participants noticed the similarities between the pairs of race-interchangeable faces or whether there was anything odd about the way the faces looked – none reported noticing the similarities between the faces of different races before it was pointed out to them.

#### Design

The order of the trials was randomized between participants. The dependent variable was the accuracy of responses in determining whether the eyes were identical between the study face and the test face. The key independent variable was whether the internal features were presented in the Black racial context or the White racial context. A second independent variable was whether the face was presented with the top and bottom halves aligned or misaligned. Whether the eyes were the same or different between the study face and test face was an independent variable. Lastly, the congruence of the mouth position (relative to the eye position) was an independent variable: if the eye position changed and the mouth position changed then this was considered congruent whereas if one feature changed and the other stayed the same then this was considered incongruent. The research ethics of the procedure was approval by the institutional review board.

### Results

Throughout the experimental research reported here, Bayesian analysis is reported in addition to the more traditional null hypothesis significance testing (NHST). Bayesian analysis is considered by some people to be superior to NHST because it is not based on the evaluation of *p*-values that are often interpreted incorrectly (e.g., the probability that the null hypothesis is true, see Rouder et al., 2009). Bayesian analysis can be used to explore evidence for the null hypothesis and the experimental hypothesis given some prior knowledge. The Bayes Factor (*BF*_10_) provides the likelihood ratio of the experimental hypothesis being true compared to the null hypothesis being true (Dienes, 2011). As such, Bayesian analysis can provide evidence for the null hypothesis being true, when using suitable priors, and so is useful in the current experiments where one is looking for the presence or absence of a difference between conditions.

The Bayes Factor does not have a set evidential cut off like the *p*-value does for significance. Instead, it reports the amount of evidence either in favor of the hypothesis or the null hypothesis as a numerical value. Some qualitative interpretation of this value has been suggested by Jeffreys (1961) such that: a Bayes Factor between 0.333 and 3 provides only anecdotal evidence; a value between 3 and 10 provides substantial evidence for the hypothesis (or for the null hypothesis if it is between 0.333 and 0.1); and a value over 10 provides strong evidence for the hypothesis (or for the null hypothesis if it less than 0.1). In the current research, this is the interpretation of the *BF*_10_ values that will be reported alongside the NHST. All Bayes Factors and NHST *p*-values were calculated using the JASP 0.7.1.12 software (Love et al., 2015).

#### Interpretation of Images

The Black faces were categorized as being a different ethnicity as the participant 85% of the time (Standard error by participant = 2.5%) and the White faces were categorized as being a different ethnicity as the participant 11% of the time (standard error by participant = 2%). Participants did not report noticing anything strange about the faces except that the position of the eyes and mouth moved and they did not spot that there were similarities between the faces of different races.

#### Standard or Partial Analysis

**FIGURE 5:**
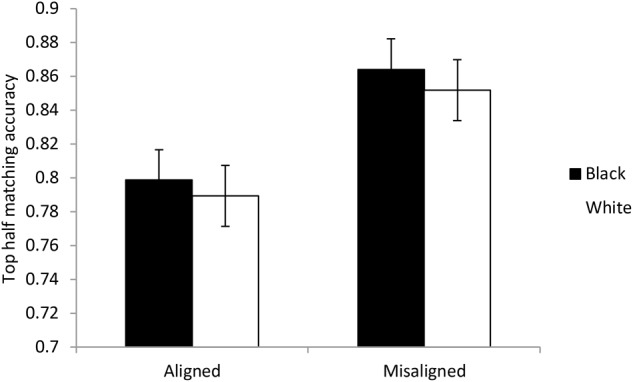
Data used for the standard analysis in Experiment 1. The figure shows the proportion of correct responses when the top halves of the faces were the same but the bottom halves were different. The data were split according to whether the faces were aligned or not and the racial context of the faces. Error bars show ± one standard error.

The method of analyzing holistic processing as advocated by Rossion (2013) looks primarily at the responses to the matching task when the top half is the same but the bottom half is different (the same-and-incongruent conditions) shown in **Figure 5**. These accuracies were used in ANOVA with factors of race and alignment. For both Black faces and White faces, misaligning the faces led to more accurate detection that the eyes were the same. Overall this effect was significant and provided very strong evidence for the hypothesis, *F*(1,53) = 19.518, *p* < 0.001, *BF*_10_ = 72,820. The effect of race was not significant and there was substantial evidence in favor of the null hypothesis, *F*(1,53) = 1.271, *p* = 0.133, *BF*_10_ = 0.274. The interaction was not significant and there was substantial evidence in favor of the null hypothesis *F*(1,53) = 0.030, *p* = 0.862, *BF*_10_ = 0.204. The effect of alignment was significant for both Black faces, *t*(53) = 3.689, *p* < 0.001, *BF*_10_ = 28.51, and White faces, *t*(53) = 3.872, *p* < 0.001, *BF*_10_ = 48.54. This demonstrates that the faces were processed in a holistic manner and the degree of holistic processing was equivalent for own-race and other-race faces.

#### Complete or Congruence Analysis

**FIGURE 6:**
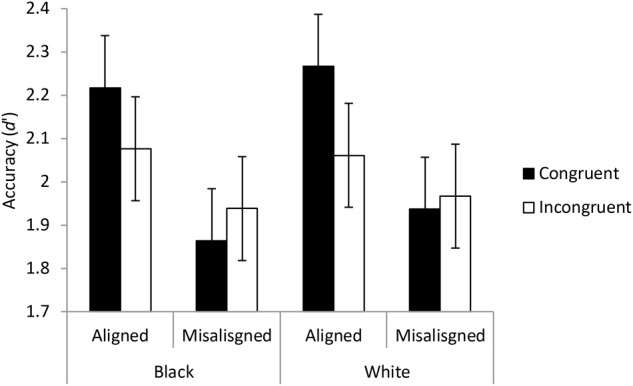
The results of Experiment 1 displayed to illustrate the complete design or the congruence design. The points show the means of the discrimination measures from the signal detection analyses for each participant based on correctly identifying that the tops of the images were the same. The error bars show ± one standard error. A higher accuracy score represented better overall performance for detecting differences and determining when the eyes were the same.

Following the method for investigating holistic processing recommended by Richler et al. (2008), discrimination scores were calculated for the detection of the eyes being the same. These scores were calculated using signal detection theory (Green and Swets, 1966) for the race-by-alignment-by-congruence conditions for each participant. Means and standard errors are shown in **Figure 6**.

A three-way ANOVA was conducted on discrimination scores with factors of race, congruence and alignment. Given that holistic processing should be revealed through a congruency-by-alignment interaction (Richler and Gauthier, 2014), we would anticipate that this interaction would interact with race in a three-way interaction if differential amounts of holistic processing were engaged in for own- and other-race faces. The three-way interaction (between race, alignment and congruence) was not significant with substantial evidence for null hypothesis, *F*(1,53) = 0.153, *p* = 0.684, *BF*_10_ = 0.182. The interaction between congruence and alignment was significant with strong evidence for the hypothesis that there is holistic processing, *F*(1,53) = 7.974, *p* = 0.007, *BF*_10_ = 46.168. This interaction shows that the congruence advantage is greater for aligned faces than for misaligned faces, which is the standard finding indicating holistic processing. The interaction between race and congruence was also significant with substantial evidence, *F*(1,53) = 1.7597, *p* = 0.008, *BF*_10_ = 5.821. This interaction shows that, if one ignores alignment, the congruence effect is greater for White faces than for Black faces. The interaction between race and alignment was not significant with substantial evidence for the null hypothesis, *F*(1,53) = 1.253, *p* = 0.268, *BF*_10_ = 0.264. The main effects showed substantial evidence for the null effect of race *F*(1,53) = 0.004, *p* = 0.952, *BF*_10_ = 0.106, but there were significant effects of congruence, *F*(1,53) = 18.740, *p* < 0.001, *BF*_10_ = 595.760, and alignment, *F*(1,53) = 18.509, *p* < 0.001, *BF*_10_ = 3236.300.

### Discussion

There was a clear composite-face effect. In the standard analysis, this was revealed by the effect of misaligning the faces on the same decisions when the bottom halves were different. This was revealed in the complete analysis by the superior performance in the congruent condition than in the incongruent condition for aligned when compared to the misaligned faces. The repositioning of the mouth, therefore, had a detrimental effect on the processing of the eyes in the whole faces. The simplest explanation for this is that the faces are being processed in a holistic manner such that there is integration between the different features of the face. The changes in the faces here are smaller than in typical composite-face experiments and so the strength of the composite-face effect is a clear indication of the importance of holistic processing in face recognition.

The more contentious issue is whether there is holistic processing for faces that are judged to be of another race. Here it is demonstrated using the comparison with the misaligned faces that the degree of holistic processing is equivalent between faces seen as being own-race and faces being seen as other-race. That is, the race-by-alignment-by-congruence interaction was not significant and, further, the Bayesian version of the analysis was used to provide substantial support that there is no three-way interaction. Further, the standard analysis shows consistent misalignment effects across different perceived races. These analyses provide support for the conclusions of Horry et al. (2015) that other race-faces are processed just as holistically as own-race faces.

Analysis of the data looking at simple congruence effects, however, reveals that the levels of performance for the own- and other-race faces are not completely equivalent. There is a clear congruence-by-race interaction on discrimination. Indeed, if the measure of holistic processing were the size of the congruence effect for aligned faces, ignoring the misaligned faces, (as used by Richler et al., 2009b, or Wyer et al., 2015) then one could argue that race has an effect on holistic processing. It can be argued that this simple congruence effect is a particularly powerful measure of face processing because it employs only whole faces – other measures of holistic processing are dependent on performance on the artificial looking misaligned images.

It is possible to reconcile the findings of a race effect on simple congruence effects but no race effect on the congruence-by-alignment interaction. The explanation comes from the fact that there may be many types of configural processing and that holistic processing is just one type. Configural processing is any processing of a face that is not done by comparing individual features. Maurer et al. (2002) describe three types of configural processing. The first is first-order relations that describe the relative position of features (e.g., eyes above nose above mouth). These relations are important in the detection of faces and understanding their orientation. As such, these are not relevant to the current discussion. The second is holistic processing which involves the gluing together of features into a gestalt. The effect of misaligning composite faces is a clear demonstration of the role that holistic processing plays in face recognition as demonstrated here and many other places. The third type of configural processing is second-order relational. This refers to analysis of the relative position facial features to each other and it has been shown that these types of changes are particularly vulnerable to the effects of inversion (Leder and Bruce, 2000). As this form of processing is related to the complex relations between facial features, it would be expected that it could produce a congruence effect with changes in the bottom of a face influencing the perception of changes in the top of the face. The current findings can be explained by the suggestion that congruence-by-alignment effect is a result of holistic processing and this form of processing is not affected by the perceived race of the face. Further, the simple congruence effect is a result of second-order relational processing and this is affected by the perceived race of a face. This argument will be returned to in the Section “General Discussion.”

Given the way the stimuli were constructed, the second-order relational features were identical between the faces perceived as Black and those perceived as White. Consequently, the difference in the processing of the faces must have been a consequence of different strategies of processing rather than difficulties with the specific features themselves. These different strategies may have been a consequence of non-expert style of processing being used for the other-race faces. This is further investigated in Experiment 2.

## Experiment 2

The majority of the experiments exploring the face composite effect have looked at matching of the top halves of the faces while the bottom halves act as to produce congruent or incongruent changes over the whole face. One reason for this maybe that the top half of the face is most important for recognition in White participants (Davies et al., 1977; Sheperd et al., 1981; Gosselin and Schyns, 2001) but what studies there are show that there is a, albeit reduced, face composite effect when matching the bottom halves of faces (Young et al., 1987; Ramon et al., 2010). One explanation for the smaller effect is that people naturally fixate on the upper region of a face (Bindemann et al., 2009) and so asking to evaluate the bottom half of the face alters the expert processing styles.

In Experiment 1 it was speculated that the difference in the congruence effects for other race faces was due to the loss of expert processing that one typically does with own-race faces. Given White participants naturally attend the eyes when viewing own-race faces (Hills and Pake, 2013), changing the area of focus will also lead to a change in processing style. If this is the case, then studying the bottom halves of the faces in a composite-face task will lead to a reduction in race-by-congruence interaction as both own-race and other-race faces will be processed in a non-typical way. Experiment 2 investigated the composite-face task when looking for changes in the bottom halves of the faces using faces perceived as own-race and other-race. Race effects on the congruence-by-alignment and the simple congruence effects were explored.

### Methods

#### Participants

There were 55 participants who took part in Experiment 2. All participants were White and were aged 18 to 22 years. Unlike in Experiment 1, selection criteria meant that none needed to be excluded.

#### Stimuli and Procedure

The stimuli were identical to those used in Experiment 1. The procedure was very similar to that used in Experiment 1. The collection of demographic details was the same and there were the same 384 trials during the experiment, which had the same format: two sequentially presented faces that may have differed in terms of their eyes or mouth positions. The difference in this experiment was the participants were directed to decide whether the bottom half of the two images presented were identical or not. This was followed by assessing whether the participants believed the faces were of the same ethnicity as themselves or not.

#### Design

The order of the trials was randomized between participants. The dependent variable was the accuracy of responses in determining whether the bottom halves were identical between the study face and the test face. The key independent variable was whether the face was presented in the Black racial context or the White racial context. A second independent variable was whether the face was presented with the top and bottom halves aligned or misaligned. Whether the positions of the mouths were the same or different between the study face and test face was an independent variable. Lastly, the congruence of the eyes position (relative to the mouth position) was an independent variable. The research ethics of the procedure was approval by the institutional review board.

### Results

#### Interpretation of Images

The Black faces were categorized as being a different ethnicity as the participant 90.3% of the time (Standard error by participant = 1.7%) and the White faces were categorized as being a different ethnicity as the participant 10.9% of the time (standard error by participant = 2.4%). Participants did not report noticing anything strange about the faces except that the position of the eyes and mouth moved and they did not spot that there were similarities between the faces of different races.

#### Standard or Partial Analysis

**FIGURE 7:**
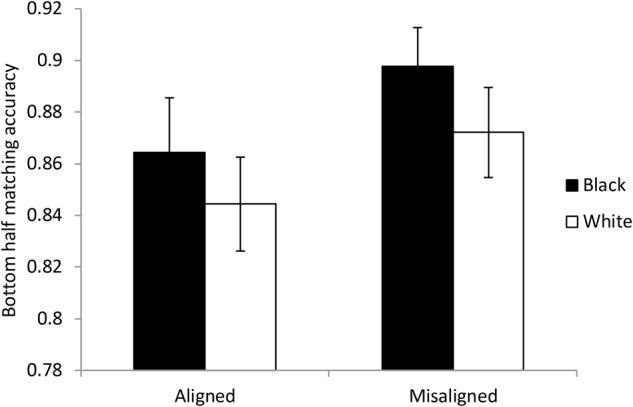
Data used for the standard analysis in Experiment 2. The figure shows the proportion of correct responses when the bottom halves of the faces were the same but the top halves were different. The data were split according to whether the faces were aligned or not and the racial context of the faces. Error bars show ± one standard error.

The method of analyzing holistic processing was the same as that advocated by Rossion (2013) but in this case focuses on responses to the matching task when the bottom half is the same but the top half is different (the same-and-incongruent conditions) shown in **Figure 7**. As the figure shows, the level of performance is higher than for comparing the top halves but it is not at ceiling. These accuracies were used in ANOVA with factors of race and alignment. For both Black faces and White faces, misaligning the faces led to more accurate detection that the bottom halves were the same when the top halves were different. Overall this effect was significant and provided very strong evidence for the hypothesis, *F*(1,54) = 10.721, *p* = 0.002, *BF*_10_ = 65.318. The effect of race was not significant and there was anecdotal evidence in favor of the null hypothesis, *F*(1,54) = 2.902, *p* = 0.094, *BF*_10_ = 0.386. The interaction was not significant and there was substantial evidence in favor of the null hypothesis *F*(1,54) < 0.001, *p* = 0.999, *BF*_10_ = 0.180. The effect of alignment was significant for both Black faces, *t*(54) = 2.514, *p* = 0.015, *BF*_10_ = 5.089, and White faces, *t*(54) = 2.412, *p* = 0.019, *BF*_10_ = 4.088. In summary, this analysis demonstrates that the faces were processed in a holistic manner and the degree of holistic processing was equivalent for own-race and other-race faces.

#### Complete or Congruence Analysis

**FIGURE 8:**
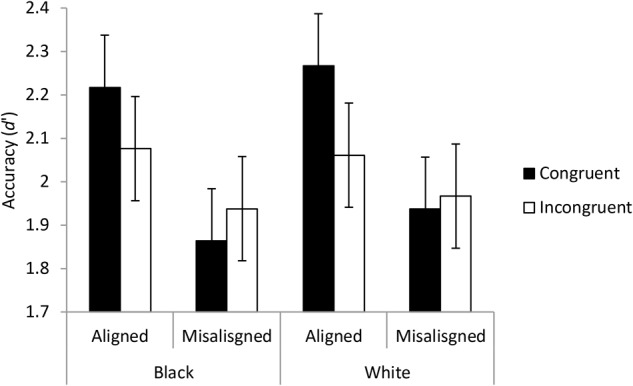
The results of Experiment 2 displayed to illustrate the complete design or the congruence design. The points show the means of the discrimination measures from the signal detection analyses for each participant based on correctly identifying that the bottoms of the images were the same. The error bars show ± one standard error. A higher accuracy score represented better overall performance for detecting differences and determining when the mouth and nose were the same.

Following the method for investigating holistic processing recommended by Richler et al. (2008) discrimination scores were calculated for the correct detection of the mouth and nose being the same as being a hit and the correct detection of the eyes being different as being a correct rejection. Means and standard errors are shown in **Figure 8**.

A three-way ANOVA was conducted on discrimination scores with factors of race, congruence and alignment. The three way interaction (between race, alignment, and congruence) was not significant with substantial evidence for null hypothesis, *F*(1,54) = 0.013, *p* = 0.908, *BF*_10_ = 0.188. The interaction between congruence and alignment was significant with substantial evidence for hypothesis, *F*(1,54) = 8.754, *p* = 0.005, *BF*_10_ = 3.892. This interaction shows that congruence advantage is greater for aligned faces than for misaligned faces. Unlike in Experiment 1, the interaction between race and congruence was not significant with substantial evidence for the null hypothesis, *F*(1,54) = 0.409, *p* = 0.525, *BF*_10_ = 0.191. The interaction between race and alignment was not significant with substantial evidence for the null hypothesis, *F*(1,54) = 0.167, *p* = 0.684, *BF*_10_ = 0.133. The main effects showed substantial evidence for the null effect of race *F*(1,54) = 0.624, *p* = 0.433, *BF*_10_ = 0.139, and the null effect of congruence, *F*(1,54) = 1.981, *p* < 0.165, *BF*_10_ = 0.258, but alignment did show a significant effect with strong evidence, *F*(1,54) = 23.812, *p* < 0.001, *BF*_10_ = 61948.241.

### Discussion

Experiment 2 demonstrates that holistic processing is taking place when participants are asked to focus on the lower part of the face as indicated by the congruence-by-alignment interaction in the congruence analysis and in the effect of misalignment in the standard analysis. As in Experiment 1, the size of this congruence-by-alignment interaction was not moderated by the perceived race of the faces and so it indicates that the amount of holistic processing is equivalent across races. The size of this congruence effect was smaller than in Experiment 1 but it is difficult to interpret such differences as direct comparisons are not meaningful because the changes made to the two halves are not comparable: The changes made were arbitrary in terms of its detectability as indicated by better overall performance in Experiment 2 than Experiment 1. That is, the change was just more noticeable in Experiment 2 than it was in Experiment 1.

The important finding in Experiment 2 was that there was no race-by-congruence interaction. That is, the size of the congruence effect was unaffected by the perceived race of the face – a finding that was different to that in Experiment 1. This is unlikely to be a result of a ceiling effect as performance was overall slightly better for Black faces than for White faces and so there was more scope for the performance on the White faces to improve. Above it was speculated that the race-by-congruence interaction observed in Experiment 1 was due to different degrees of second-order relational information being processed for other-race faces than for own-race faces. If this is the case then it appears that when participants are directed toward lower parts of faces, this difference in the processing of second-order relational features disappears. This is consistent with there being an expertise advantage in second-order relational processing for own-race faces only when viewed naturally, but forcing a different viewing pattern interrupts this expertise (Hills et al., 2013). This loss of expert-style processing reduces the simple congruence effect overall and means that the own-race advantage in second-order relational processing disappears – because both own-race and other-race faces are being processed in a non-expert manner.

Speculation can be made concerning what would happen if this experiment were carried out with people who do not typically look at the top half of faces. If people were to routinely look at the lower half of the faces then the pattern of results across the two experiments would be expected to be reversed. Carroll and Chang (1970) suggest that the lower halves of Black faces are more diagnostic of identity than the upper halves and so it might be expected that Black participants focus more on the lower half of the faces. This would suggest that, as the vast majority of research in the composite face effect uses the top halves of faces, the entire field has potentially displayed a White bias by focusing on the top halves of faces. The current research equally has this White bias that could be addressed in future research.

## General Discussion

Previous research into race effects in the composite-face tasks has provided inconsistent findings. Some studies have shown that race does change the size of the composite-face effect (e.g., Michel et al., 2006) whereas other have shown that the effect is unaffected by race (Harrison et al., 2014). The results from the experiments reported here provide a similar variety of outcomes. Experiment 1 showed an effect of race on simple congruence effects but no effect on congruence-by-alignment interactions whereas Experiment 2 showed no race effects at all, when White participants were tested on faces that were perceived as either being Black or White.

The current findings, as well as the previous ones, can be resolved by considering configural face processing to be split up, as described by Maurer et al. (2002), into holistic processing and second-order relational processing (note that Richler et al., 2012, describe similar multiple mechanisms of face processing but their terminology is slightly different). Holistic processing is the gestalt aspect based on template matching of faces and this is disrupted by misalignment of the two halves of the face. A loss in the effect of congruence caused by misalignment shows that holistic face processing is responsible for the congruence effect when the face is aligned and presented as a whole. The current findings that this loss of congruence is equivalent for own-race and other-race faces suggests that holistic processing is used for both own-race and other-race faces. Further, Bayesian analysis shows that there is substantial evidence that the amount of holistic processing is the same across faces of different races providing direct support that other-race faces are processed just as holistically as own-race faces.

The second-order relational processing of features concern the relative position of features and this is what was varied in the current experiments. Ignoring the effects of misalignment, changing the relative position of features on one half of the face affects detection of changes on the other half – that is the simple congruence effect. Experiment 1 showed that the size of this effect was affected by the perceived race of the faces when viewed normally (that is focusing on the top half of the face). The congruence effect was smaller for other-race faces indicating that these faces were processed with less second-order relational processing than own-race faces. In Experiment 2, there was no effect of race on the simple congruence effect, which is interpreted as second-order relational processing being equivalent in for both own-race and other-race faces when they are viewed in a non-typical manner. The proposal is that second-order relations features are processed less in non-expertly processed faces. This non-expert processing happens when either a face is from an unfamiliar group or if a person is directed to look at a face in an unusual way.

The question remains as to why second-order relational information is processed less for other-race faces than for own-race faces. Given the way the stimuli were generated, the second-order relational features were identical between the faces perceived as own-race and those perceived as other-race. This means that any explanation based on there being a tuning of the face processing system to the specific changes of own-race faces (e.g., Valentine and Endo, 1992) cannot account for the differences observed. What is happening is the perception of the race of the face changes the way the second-order relational features are processed even though the internal features are identical between the sets of stimuli. This suggests a shift takes place from expert processing of own-race faces to non-expert processing of other-race faces guided by the racial context of the image. This could be something as simple as directing attention to lower facial features if the other-race face is Black, for example, or it may be a more subtle change in processing style. This change in processing style for other-race faces is consistent with the socio-cognitive explanation of the own-race bias by Sporer (2001) where the in-group faces are processed automatically more deeply than out-group faces. Part of this automatic deeper processing may involve deeper processing of second-order relational features. The evidence from Experiment 2 supports the idea that the change in the congruence effect is a result of expert processing of only own-race faces because shifting to a non-typical viewing style disrupts expert processing even for own-race faces.

What has been shown here is that holistic processing is as much a feature of other-race face processing as for own-race face processing but there are differences in the processing of second-order relational features, at least for White participants. The claim being made here is that there are differences in the processing of faces of different races based on their perceived race but this does not necessarily mean that own-race bias is being driven by a loss of second-order relational information. What is being concluded is that merely looking like a face from another race is sufficient to change how deeply a face is processed. This change of depth of processing may be responsible for the changes in second-order relational processing observed here. Burton et al. (2015) argue that second-order configural features may have a remarkably unimportant role in face recognition and so observing a change in this processing type across races can only partially help to explain the own-race bias in recognition observed elsewhere (e.g., Meissner and Brigham, 2001). Maybe the lower second-order configural processing is covariate of the cause of the own-race bias rather than being the cause of this difference in recognition ability. The second-order configural features cannot be the whole story as Rhodes et al. (2006) pointed out, part-based feature processing is also affected by race of the face – although, in that study the different races of faces had different facial features and so the effect might not be independent of familiarity effects. Future research could use the race interchangeable faces developed here to isolate the perceived-race effect from the race-of-features effect in the race effects observed in changing parts of the faces.

## Conclusion

The present research used images in which the internal features could be matched across racial contexts and manipulated independently from the perceived race of the faces. Analysis of the composite-face effect with these faces suggests that the degree of holistic encoding that takes place in face processing, as indicated by the congruence-by-alignment effect, is unaffected by the perceived race of the faces. The simple effect of congruence, however, was affected by the perceived race of the faces (even though the internal features were matched across races) and it is argued here that this is a result of there being stronger second-order relational processing of own-race faces than other-race faces. This demonstrates that the race of the face actively influences the nature of facial processing used leading to shallower processing of faces perceived as being other-race. The own-race bias in face recognition may be a consequence of a lack of configural processing but this would be second-order relational processing and not the holistic processing it has previously been suggested to be.

## Ethics Statement

This study was carried out in accordance with the recommendations of the British Psychological Society. The protocol was approved by the School of Psychology Research Ethics Committee, Cardiff University. All subjects or participants gave written informed consent in accordance with the Declaration of Helsinki.

## Author Contributions

ML designed, ran and analyzed the experiments, and drafted the manuscript. PH contributed to the theoretical development and drafting of the manuscript.

## Conflict of Interest Statement

The authors declare that the research was conducted in the absence of any commercial or financial relationships that could be construed as a potential conflict of interest.
